# Cross-sectional Study on the Impact of Discount Pricing and Price Competition on Community Pharmacy Practice

**DOI:** 10.7759/cureus.9903

**Published:** 2020-08-20

**Authors:** Allan Mathews, Long C Ming, Farid Z Che Rose, Syed A Abbas

**Affiliations:** 1 Faculty of Pharmacy, Quest International University Perak, Ipoh, MYS; 2 PAP Rashidah Sa’adatul Bolkiah Institute of Health Sciences, Universiti Brunei Darussalam, Gadong, BRN; 3 Faculty of Science and Technology, Quest International University Perak, Ipoh, MYS

**Keywords:** community pharmacy, discount pricing, competition, medicine pricing

## Abstract

Background

Without stipulated legislation, a free pricing policy can lead to a disparity in prices among private healthcare setups. Competition is especially rampant among community pharmacies, especially in the Sabah state of Malaysia, where the recent years have witnessed the steady growth of pharmacy players from Peninsular Malaysia. Thus, this study aimed to examine the impact of price competition and discount pricing on the practice of community pharmacy in Sabah, Malaysia.

Methods

This was a cross-sectional study using an online questionnaire. Survey participants included community pharmacists practicing in Sabah. The validated and pilot-tested questionnaire consisted of three parts: background information of the pharmacy, attitudes and perception toward medicine prices, and practice of discount pricing. All required data were collected from community pharmacists practicing only in Sabah. Data were then analyzed by using descriptive, Chi-Square, and Kendall's tau-b tests.

Results

Of the 150 community pharmacists contacted, only 70 responded, providing a response rate of 47%. In terms of pharmacy type, 71% of the respondents were pharmacist-owned independent pharmacies, while 19% were pharmacy chains owned by community pharmacists. The remaining were pharmacies owned by non-pharmacists (10%). Sixty percent of the community pharmacies had been in existence for more than 10 years, with 12% in existence for less than two years, and 28% in existence for three to 10 years. More than 80% of the respondents stated that the business aspect of community pharmacy had overwhelmed the professional practice aspects and that community pharmacists have become providers of products instead of providers of care. In terms of professionalism, 87% also noted that they are being perceived as profiteering in the medicine business at the expense of patients.

Conclusions

The free market situation in Malaysia for medicine pricing has brought a detrimental consequence for community pharmacists with each one trying to undercut prices. Differing pricing mechanisms of medicines based on the quantity ordered contribute to the problem of discount pricing and price competition. Most community pharmacists, as indicated by this study, want the problem to be addressed.

## Introduction

Community pharmacy in Malaysia has evolved over the last five decades, hastened by the annual output of approximately 1200 pharmacy graduates [1]. Like most developing countries, medicine-dispensing practices based on prescriptions are not popular, since the practice of dispensing by community pharmacists and a national health scheme are not in place. Laws governing the different classes of medicines are based on the Poisons Act of 1952. This law divides all medicines into two main categories: controlled medicines and over-the-counter (OTC) medicines. OTC medicines are freely available in the market (e.g., in supermarkets). Controlled medicines are further divided into those requiring a prescription and those which the community pharmacist can supply directly to the patient [1].

This practice has evolved to where the focus is on direct medicine pricing, thus creating pricing competition. This is in stark contrast with emphasis by the World Health Organization Pharmacy Chapter on patient care and medication safety [2]. The current free market situation has been unable to control medicine prices, and pharmacies tend to reduce prices to attract customers. Challenges, including market competition, legislative issues, and customers' expectations, distract community pharmacists from delivering professional services such as dispensing and counseling [2]. The general perception is that pharmacies are like any other retail industry and are subject to normal competition, making the shift to a patient-focused service more challenging as well as resulting in non-compliance to ethical practice [3]. Differing pricing and bonus scheme along the distribution chain add to the problem, leading to distortion in the market and consumers who are confused about the actual price of a drug [3]. Professionalism of practice is compromised in Malaysia, as evidenced by a study in 2001, which noted the need for aggressive efforts to improve this image [4]. The Malaysian Community Pharmacy Guild noted the need to standardize prices of medicines and the need to regulate prices, as disparity exists [5].

The transition of healthcare system financing in many countries around the world had a direct effect on the utilization of pharmaceuticals by population. Within this context, in the majority of countries, spending on pharmaceuticals is rapidly escalating when compared with an increase in healthcare expenditure [6]. In return, the majority of healthcare systems are unable to fulfill the ever-increasing demands of pharmaceutical care. Also, scarcity of resources, higher medication prices, and limited addition of new medicines made it more challenging to muddle through the demand and supply of pharmaceuticals. A systematic review in 36 countries revealed that retail mark-ups for medicines ranged from 10% to 552%, which made it exceedingly difficult for consumers to purchase and use medications [7].

Shifting the concerns of "affordability" to the Malaysian context, medicine prices escalated much faster as compared to other developed countries. A proportionate increase of 7% to 28% in medicine prices was reported between 1990 and 1992, whereas prices in the United Kingdom remained constant during the same period [8]. Furthermore, the median price ratio (MPR) for innovator brand medicines was 16.35 times higher than the international reference price (IRP) in Malaysia [9]. Also, the MPR for the most commonly sold generic medicines was 6.89 times higher than the IRP [9]. Similar to what was reported earlier, the mean retail medicine prices in Penang, Malaysia, were 30.3% to 148.2% higher than the mean retail drug prices in Australia [10].

The higher medicine prices in Malaysia are firmly attributed to the absence of price control and regulations in the country. Medicine manufacturers, distributors, and healthcare practitioners, therefore, can set their own price for pharmaceuticals. In addition to the lack of price regulation, another factor related to higher medicine prices is connected to increased mark-ups by healthcare professionals [11]. The "personal prices" of medicines and higher mark-up rates have created intense competition among healthcare providers around the country [12]. The competition intensifies as most Malaysian consumers prefer to buy medicines from community pharmacies due to long waiting times and lack of certain medicines at public healthcare institutes [13]. However, even in the presence of such extensive competition, a big price disparity is consistently observed among Malaysian private healthcare settings. The absence of price regulation again plays a key role in establishing the price disparity as it usurps the market share, thus diminishing the survival of the weak players [14]. This is consistent with reports from healthcare settings in Malaysia-that the price disparity has reduced their profit margin and hence is a threat to their survival. Furthermore, the price disparity has undermined the dignity and professionalism of healthcare settings in Malaysia [15,16].

The price disparity is due to the absence of drug price regulation in Malaysia and pharmaceutical companies, wholesalers, and healthcare providers in the private sector can set their own retail selling price. Previous studies on price differences were confined to qualitative surveys or were between general practitioner clinics and registered pharmacies. They were mainly carried out in small populations and there is inadequate knowledge in price competition and discount pricing. There is a paucity of insights on pharmacists' views on the medicine pricing saga and practice [17].

The aims of this study were to explore the impact that price competition and discount pricing have on the practice of community pharmacy in Malaysia [9,18]. The study focused on one state where a pricing strategy was implemented. It is assumed that Sabah, being part of Malaysia and subject to the same legislation and similar business environment, would reflect general conclusions that can be drawn. The findings of this study could portray an accurate picture of medicine price disparity, providing a foundation for the development of drug price policies at national and state levels.

Pricing of medicines is of utmost importance to patients and price disparity causes patients to lose confidence in the system. The pricing strategy should aim to ensure that the patient pays the same price for the same brand of medicine wherever it is obtained. The current practice to incentivise big purchasers by offering bonuses contributes to price disparity due to them being able to bonus-net the prices. The control of prices at the consumer level will contribute to a more level playing field and competition shifts towards service levels.

## Materials and methods

Research instrument

A 20-item questionnaire was developed to explore the views of community pharmacists practicing in Sabah, Malaysia, on the price and discount culture and how these factors are affecting professional practice. A five-point Likert scale was used. Pharmacy experts and a statistician from the Quest International University validated and yielded a Cronbach alpha score of 0.72.

Study design, settings, and participants

This study was designed as a cross-sectional e-survey sent to 150 community pharmacists. Ethical approval was received from the Research, Post-graduates Studies, and Strategic Linkages Committee of the Faculty of Pharmacy at Quest International University in Ipoh, Perak. Participants were community pharmacists practicing in the Sabah. The Sabah population is approximately 10% of the total population of Malaysia and is situated on the island of Borneo, separated by the South China Sea from Peninsular Malaysia. All community pharmacists in the state were sent the questionnaire and e-consent form, which was completed online. The Sabah Pharmaceutical Society, which had the database, sent out the questionnaire to all community pharmacists in Sabah and obtained the responses.

Statistical analysis

IBM SPSS Statistics for Windows, Version 25.0 (Armonk, NY: IBM Corp.) was used for data analysis. Descriptive, Chi-Square, and Kendall's tau-b tests were used. Kendall's tau-b is a non-parametric measure of the strength and direction of association that exists between two variables measured on at least an ordinal scale. We adopted Chi-Square test to investigate the independency between the items. The null hypothesis of Chi-Square test is that no relationship exists among the items. P≤ 0.05 was considered to be statistically significant.

## Results

Seventy community pharmacists responded to the survey, providing a response rate of 47%. A total of 71% of the respondents were pharmacist-owned independent pharmacies, while 19% were pharmacy chains owned by community pharmacists. The remaining were independent pharmacies owned by non-pharmacists (7%) and chain pharmacies owned by non-pharmacists (3%). Sixty percent of the community pharmacies had been in existence for more than 10 years, with 12% in existence for zero to two years and 28% in existence for three to 10 years. Respondents' data were tabulated and is shown in Table 1, and the responses to the questions are presented in Table 2.

**Table 1:** The ownership of pharmacies and years in existence (n=70) of respondents

	Characteristic	%
Ownership (%)	Pharmacist-owned independent pharmacy	71
	Pharmacist-owned pharmacy chain	19
	Non-pharmacist-owned independent pharmacy	7
	Non-pharmacist-owned pharmacy chain	3
Years of operation	0-2 years	12
	3-5 years	14
	6-10 years	14
	>10 years	60

**Table 2:** Community pharmacist’s responses to study questions 1 – Strongly Disagree          2 – Disagree          3 – Neutral            4 – Agree               5 – Strongly Agree MSP – minimum selling price

Question No.	Indicator	%				
		1	2	3	4	5
1	The Business Aspect has overwhelmed the Professional Aspects of Community Pharmacy Practice.	4.3	2.9	10.0	51.4	31.4
2	Due to the Discount Culture, the community pharmacists have evolved into being providers of products and not providers of care.	1.4	5.7	12.9	45.7	34.3
3	The focus on the price of medicines has brought about a situation where community pharmacists are perceived as profiteering in the medicine business at the expense of patients.	2.9	4.3	5.7	52.9	34.3
4	The Discount Culture has brought about a negative and demeaning impact on professional Community Pharmacy Practice.	1.4	0.0	1.4	42.9	54.3
5	There is NO need to address the Discount Culture for Dispensed Medicines as it is a free market environment and leave it to the market forces to determine the price of medicines.	1	2	3	4	5
		24.3	44.3	11.4	15.7	4.3
6	Medicines should be considered as any other commodity e.g. chocolates.	55.7	32.9	4.3	5.7	1.4
7	The implementation of a Professional Fee for community pharmacists for services rendered would assist in addressing the Discount Culture.	2.9	18.6	28.6	31.4	18.5
8	Fixing of prices of Dispensed Medicines at the consumer level would assist in addressing the Discount Culture even though this is against the Competition Act.	1.4	8.6	15.7	55.7	18.6
9	The concept of Recommended MSP for Group C Poisons addresses the aspect that community pharmacists are professionals and not just traders.	1.4	5.7	7.1	45.7	40.1
10	Every patient that the community pharmacist decides to supply a Group C Poison will be based on the condition presented which can be simple or complex, hence needing varying amounts of time and knowledge.	0.0	0.0	0.0	50.0	50.0
11	The Discount Culture has caused patients to wonder whether pharmacists have been overcharging all this while and many patients have switched to other pharmacies.	0.0	1.4	5.7	42.9	50.0
12	Due to the Discount Culture much professional goodwill has been lost amongst long-established loyal patients and customers.	0.0	0.0	0.0	45.7	42.9
13	Price Capping of Dispensed Medicines will NOT address the Discount Culture and Price War will persist.	2.9	21.5	18.6	38.5	18.5
14	The Discount Culture is perpetuated by the NEWER community pharmacists for survival.	4.3	0.0	17.1	45.7	12.9
15	The Recommended MSP addresses the margin needed to manage the product e.g. operations, storage, ordering, checking, etc.	0.0	4.3	7.1	65.7	22.9
16	The Recommended MSP incorporates a service margin which is not fixed but can be higher depending on the level of service provided.	0.0	2.9	17.1	65.7	14.3
17	Recommended MSP has been implemented in Sabah for the last 5 years or so and it has assisted me in my practice and price haggling has been reduced.	12.9	25.7	27.1	30.0	4.3
18	I have charged more than the recommended MSP since the patient is happy with the level of service.	7.1	10.0	38.6	41.4	2.9
19	I have most of the time charged less than the recommended MSP.	11.4	37.1	28.6	15.7	7.2
20	The main reason I charged less or would charge less than the recommended MSP is because the patient indicated that the other pharmacy is charging so.	2.9	5.7	25.7	37.1	28.6

More than 80% stated that the business aspect of community pharmacy had overwhelmed the professional practice aspects and that community pharmacists have become providers of products rather than providers of care. Eighty-seven percent also noted that they are perceived as profiteering in the medicine business at the expense of patients.

More than two-thirds disagreed with the notion that nothing needs to be done with price competition and discount culture since it is a free market situation. However, 20% agreed with this notion. Seven percent stated that they consider medicines to be like any other commodity (e.g., chocolates). Approximately 58% stated that the problem was perpetuated by newer community pharmacists for the sake of survival.

About half of the respondents noted that having a professional fee for services rendered, which involves time, knowledge, and skill, will assist in addressing the price competition and discount pricing, although about one-fifth of respondents disagreed.

More than 85% also noted that having a minimum selling price (MSP) will help in addressing the issue. More than 80% also supported the notion that the MSP will address margins relating to product management as well as for services rendered. However, the effectiveness of the MSP after implementing it for more than five years gave mixed results - approximately 34% stated it has assisted in addressing the discount culture and price, while 38% noted it has not. Meanwhile, 44% said that they had charged more than the MSP as patients were happy with the services rendered. Approximately 48% disagreed that they have charged less than the MSP, and the majority reason given was that the other pharmacy was pricing less than the MSP.

More than 85% of respondents also stated that the discount culture had affected patient loyalty and goodwill. To address this problem, more than half of the respondents noted that price capping or fixing the ceiling price would not address the problem. On the other hand, nearly a quarter responded that it would.

The aspects/areas that are significantly associated are presented in Table 3. The majority of respondents noted that business aspects of community pharmacy had overwhelmed the professional aspects, and there was a statistically significant correlation to community pharmacists being providers of products rather than providers of care. They also noted that community pharmacists are perceived as profiteering at the expense of patients, which had a significant correlation to loss of loyalty of patients by the Chi-Square test (Table 3).

**Table 3:** Association between different pricing aspects *denotes statistical significance at 5% MSP - minimum selling price; RMSP - recommended MSP

Aspects/ Question Number	Chi-Square	Kendall’s tau-b
Business aspect versus discount culture/ 1 versus 2	0.0010*	0.0000*
Business aspect versus profiteering/ 1 versus 3	0.0270*	0.0330*
Price standardization versus recommended MSP/ 8 versus 9	0.0000*	0.0030*
Profiteering versus over-charging/ 3 versus 11	0.0360*	Not significant
Negative connotation of discount culture versus over-charging / 4 versus 11	0.0080*	0.0000*
Business aspect versus loss of professional goodwill / 1 versus 12	Not significant	0.0220*
Discount culture versus loss of professional goodwill/ 2 versus 12	Not significant	0.0010*
Over-charging versus loss of professional goodwill / 11 versus 12	0.0000*	0.0000*
Lower than RMSP versus price comparison/ 19 versus 20	0.0470*	0.0500*

## Discussion

There was a significant correlation between handling the discount culture and price competition by having price capping or MSP and that this would address the perception of community pharmacists being professionals and not just traders. The discount culture has negatively impacted the professional community pharmacy practice, and this is associated significantly with the perception of patients that they have been overcharged.

The over-emphasis on business aspects leading to community pharmacists being providers of products rather than care is correlated significantly (Kendall's tau-b) with the loss of loyalty among patients. The significant reason for charging less than the MSP is in response to other pharmacies doing the same.

Community pharmacy practice varies significantly from country to country. European community pharmacy has shifted toward a patient-focused profession [19]. There are differing remuneration schemes in different countries. For example, in Croatia, services are not remunerated, whereas in Denmark, they are, and medicine prices are fixed, as is the case in Finland, but not in Norway, where maximum prices for prescription-only medicines are set [20]. In England, medicine pricing is determined by the government, as is the case in Germany, which defines where competition is based on quality and service [21]. Pricing in Spain is set by the government's national health system, but there are few paid services [21]. Dispensing of medication remains the core activity in Ireland, although expanded services have taken place, such as in Portugal, where generic substitution is remunerated, but cost-containment has occurred [21]. In Sweden, which is a welfare state that determines retail margins for all pharmacies and allows generic substitution for the cheapest option [22]. In Switzerland, the dispensing function is predominant, but there is counseling as well as vaccination [23]. In Australia, there is a change from dispensing to professional services [24]. In the United States, there is generic prescription pricing competition and a lack of remuneration, unlike in Alberta, Canada, where remuneration is for direct patient-related services [25].

Services provided can range from the traditional role in the dispensing of medicines based on prescription issued by a medical, dental, or veterinary practitioner to very advanced medicine management of patients, including the administration of medicines by injections. Even in countries where there is a separation of functions of dispensing and prescribing, community pharmacists have ventured into dealing with non-healthcare products, including traditional and complementary medicines, cosmetics, hats, consumer goods, with the view of increasing business sales volume [26]. This has greatly diluted professionalism in pharmacy practice. There is much discontentment among community pharmacists, with the focus on discount and price competition, rather than on providing care to patients. This unfortunate turn of events for the profession has resulted in community pharmacists being viewed as profiteering in the medicine business, resulting in loss of patients and long-established goodwill [27].

In developed countries, such as the United Kingdom and Australia, where there are health insurance schemes, prices are controlled at the consumer level, and competition is on the level of service provided. However, in Malaysia, where there is no separation of functions, the community pharmacy has become a place where price competition and discount culture are fermented by varying discounts and trade deals, allowing bigger pharmacies and chain pharmacies to buy in bulk and bring down the price dramatically. Smaller-scale pharmacies, to survive, have to slash margins. Patients shop around knowing there will be one that can provide a lower price than the other [7,28].

This study has shown that professionalism in community pharmacy practice has taken a back seat in Malaysia, which can be attributed to discount pricing and price competition. The level of professional frustration among community pharmacists can be seen from this study, as they have indicated they want something to be done to address the problem. It is interesting to note that there is a small percentage (7%) of community pharmacists who view medicines as being the same as chocolates, indicating the complete loss of professionalism. Although small in number, they damage the profession on a large scale, as the general public will ponder what is the real role of the community pharmacist.

On the positive side, about half of respondents noted that having a professional fee for services will help address the discount culture and price competition situation. In countries with dispensing separation, there is the dispensing fee, which is linked to the number of products in a prescription. However, more countries are now remunerating pharmacists for services rendered [29].

In addressing the discount culture and price competition, establishing a price control mechanism, including the MSP, is one mechanism to restore respect for the profession. However, price capping or fixing prices at the consumer level is not expected to address the problem, according to this study.

In an extremely competitive market based solely on price, this study revealed that the majority of community pharmacists affected are the newer community pharmacists who would have no choice but to match or lower prices, as compared to chain pharmacies or well-established pharmacies, for the sake of survival. Also, the Malaysian Pharmaceutical Society introduced benchmarking guidelines to raise professional standards; however, only 51% of respondents are aware of it, leading to unethical practices [30].

There were statistically significant associations between certain areas of community pharmacy practice revealed in this study. Clear associations that can be deduced include: 1) the overwhelming of professionalism in community pharmacy by the discount culture and price competition, and 2) pharmacists have become providers of products rather than care. The majority of respondents noted that patients now view them as profiteering in the medicine business, resulting in loss of loyalty. To overcome the problem, the concept of MSP or price capping was agreed upon by most respondents.

There is an urgent need for healthcare authorities to address the pricing of medicines by imposing an MSP and provide a level-playing field for community pharmacists. Competition among the community pharmacists should be based on service since the practice involves knowledge, skill, and time rather than competition based on product pricing. Price capping is deemed inappropriate since different conditions need differing levels of knowledge, skill, and time. Patients will be willing to pay for better services rendered. Evidence from the survey indicated that 44% of community pharmacists agreed that patients were willing to pay for services against 17% who did not agree. Addressing the price issue will bring back much needed public confidence in the healthcare sector.

Limitations

This study was conducted in the state of Sabah, one of 13 states in Malaysia, where a price control mechanism has been implemented for more than five years. There is an ongoing effort to further conduct a study covering the other states in Malaysia. Although the questionnaire was very carefully prepared and data collection was individualized, a hundred percent avoidance of recall bias is not possible.

## Conclusions

The free market situation in Malaysia for medicine pricing has brought a detrimental consequence for community pharmacists, with each one trying to outdo the other by lowering prices. Professionalism and good service have been set aside. Differing pricing mechanisms of medicines to different pharmacies contribute to the problem of discount pricing and price competition. Most community pharmacists, as indicated by this study, want the problem to be addressed. The study shows that having a recommended MSP will not address the problem. Establishing the MSP is one mechanism the majority of respondents agreed to. Another is the need for a professional fee for pharmacists for services rendered. There is a need for the government to step in to address the problem.
